# Development of a mobile app for the evaluation of patients with chronic rhinosinusitis^☆^

**DOI:** 10.1016/j.bjorl.2023.101375

**Published:** 2023-12-14

**Authors:** Priscila Novaes Ferraiolo, Sergio Duarte Dortas, Fabiana Chagas da Cruz, Priscilla Campos de Souza Ramos, José Elabras Filho, Marise da Penha Costa Marques, Cláudia Maria Valete-Rosalino

**Affiliations:** a Universidade Federal do Rio de Janeiro (UFRJ), Hospital Universitário Clementino Fraga Filho (HUCFF), Serviço de Otorrinolaringologia, Rio de Janeiro, RJ, Brazil; b Universidade Federal do Rio de Janeiro (UFRJ), Hospital Universitário Clementino Fraga Filho (HUCFF), Serviço de Imunologia, Rio de Janeiro, RJ, Brazil; c Universidade Federal do Rio de Janeiro (UFRJ), Faculdade de Medicina, Departamento de Otorrinolaringologia e Oftalmologia, Rio de Janeiro, RJ, Brazil; d Fundação Oswaldo Cruz, Instituto Nacional de Infectologia Evandro Chagas, Laboratório de Pesquisa Clínica e Vigilância em Leishmanioses, Rio de Janeiro, RJ, Brazil

**Keywords:** Nasal polyps, Paranasal sinus diseases, Rhinitis, Sinusitis, Asthma, mobile application

## Abstract

**Objective:**

To develop a mobile application with a standardized routine, to be used by general otolaryngologists for evaluating patients with chronic rhinosinusitis.

**Methods:**

A systematic review was made to identify outcomes, recommendations and what tests that would be used in the routine evaluation of patients with chronic rhinosinusitis; establish an expert consensus on items to be included in this routine evaluation of patients with chronic rhinosinusitis using the Delphi method; development of an application for use on a mobile device, with the routine evaluation of patients with chronic rhinosinusitis.

**Results:**

Based on the systematic review, the outcomes used in studies about chronic rhinosinusitis were identified, as well as guidelines recommendations, which showed discrepancies between them. These recommendations and outcomes were presented to specialists in chronic rhinosinusitis, until a consensus was reached. As a result of the Delphi method, the flowchart of the routine evaluation of patients with chronic rhinosinusitis was defined, and then was used for the development of the mobile application.

**Conclusion:**

The creation of the mobile application for evaluating patients with chronic rhinosinusitis followed an adequate methodology of elaboration made by specialists in the field of chronic rhinosinusitis, standardizing the investigation of these patients.

**Level of evidence:**

Level 5.

## Highlights

• Patients with chronic rhinosinusitis are a heterogeneous group.
• There are regional diferences of patients with chronic rhinosinusitis.
• Chronic rhinosinusitis international guidelines are not unanimous.
• An expert consensus can standardize the care of these patients.
• The creation of a mobile application make the expert consensus more accessible to use.

## Introduction

Chronic Rhinosinusitis (CRS) is defined, according to the European Position Paper on Rhinosinusitis and Nasal Polyps^1^ as inflammation of the nose and sinuses lasting longer than 12 weeks.

It is a highly prevalent disease, diagnosed in 12.9% of the European population^2^ and in 11.9% of the population of the United States of America.^2^ Its prevalence in the city of São Paulo is 5.5%.^3^

Another characteristic of CRS is the existence of a significant regional particularities. There are differences of CRS endotypes in different regions worldwide^4^ Furthermore it is not recommended to uncritically adopt data from other regions of the world because of these several differences.^5^

Patients with CRS are actually a heterogeneous group with similar signs and symptoms and have a higher chance of having asthma, respiratory allergy, and Nonsteroidal anti-inflammatory drug-Exacerbated Respiratory Disease (NERD).^5^ In São Paulo, the conditions most related to CRS were asthma, allergic rhinitis and low-income people.^3^

Signs and symptoms of CRS can also be a manifestation of other diseases such as: tumors, odontogenic sinusitis, fungal ball, primary ciliary dyskinesias, cystic fibrosis, Allergic Bronchopulmonary Aspergillosis (ABPA), vasculitis and immunodeficiencies, therefore being characterized as secondary CRS.^1^

The correct diagnosis of primary or secondary CRS, as well as the comorbidities and inflammatory patterns present in these patients, changes their prognosis and treatment.

The most used endotypic classification of CRS is based on the type 2 immune response, characterized by high IgE and high serum eosinophils, or non-type 2 immune response. The type 2 immune response profile tends to be more resistant to current treatments and with a high recurrence rate when compared to the non-type 2 profile. This differentiation can predict the response to different treatment modalities such as corticosteroids, nasosinusal endoscopic surgery and immunobiologicals.^6^ The diagnosis of comorbidities can predict the inflammatory profile of these patients as well as their prognosis. Those with asthma tend to have a type 2 immune response and greater recurrence of nasal polyps as well as more difficult-to-control disease.^7^

This ability to correctly diagnose the endotype of patients with CRS depends on their correct investigation.

There are still discrepancies between international guidelines on how the clinical investigation of patients with CRS should be carried out. The opinions on which tests to request, when to request, which comorbidities should be investigated and when to refer these patients to other specialists differ depending on the guideline used.^8^

Mobile health applications are emerging as novel tools for self-management in chronic respiratory diseases and can help better understanding real-life burden of CRS. Recently, a mobile application that enables self-monitoring and patient education, called mySinusitisCoach, was launched by the European Forum for Research and Education in Allergy and Airway Diseases (EUFOREA).^9^

Taking into account the regional differences in CRS and the discrepancies between guidelines recommendations, the evaluation of patients with this disease should be individualized and standardized for the studied population.

Therefore, it is necessary to develop a consensus of Brazilian experts on how to evaluate patients with CRS, that can be easily used by general otolaryngologists.

The objective of this study is o develop a mobile application with a standardized routine for evaluating patients with chronic rhinosinusitis, to be used by general otolaryngologists.

## Methods

This study was conducted in accordance with the Declaration of Helsinki. Ethical approval for this study was obtained from Instituto Nacional de Infectologia Evandro Chagas (INI/Fiocruz) ethic committee under de approval number 3.192.285, in March, 12th 2019.

The COMET initiative^10^ published a systematic review to standardize the outcomes to be used in CRS studies.^11^ Based on these outcomes, the main researcher chose which outcomes should be used in the Delphi method^11^ for: subjective assessment of symptoms; objective assessment of Nasal Endoscopy (NE) and Computed Tomography (CT) of the paranasal sinuses; which disease-specific quality of life questionnaire was going to be used and what criteria to use for assessment of adherence to treatment.

A guideline-oriented approach described by Kotter, Blozik and Schere^12^ was chosen to extract the recommendations from these guidelines and the Delphi method^13^ was used to achieve convergence of opinion in formulating the routine for evaluating patients with CRS.

Seven guidelines were selected.^1^, ^14^, ^15^, ^16^, ^17^, ^18^, ^19^

**Figure 1:**
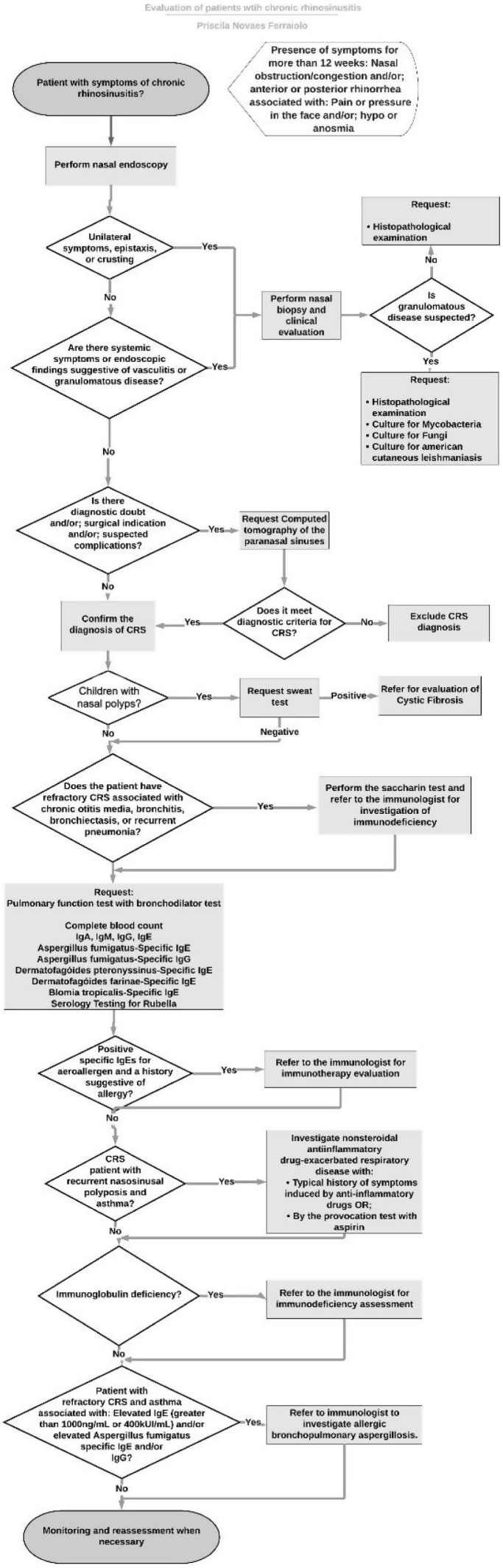
Flowchart.

Using the recommendations of the previously selected guidelines, a flowchart was formulated for the investigation of patients with CRS (Fig. 1). Differences of opinion between the guidelines were pointed out. These divergences, as well as each step in the flowchart were discussed by the panel of experts through the Delphi method.

During the Delphi method step, the outcomes, tests, and recommendations to be used in the routine evaluation of patients with CRS that were previously identified were used to formulate a questionnaire.

Seven physicians were invited. The inclusion criteria for the experts to be invited were working in the field of otorhinolaryngology or allergy/immunology, specifically with patients with the diagnosis of CRS, working in Rio de Janeiro state and in a University Hospital.

Each previously selected item was scored on a Likert scale: (1) Strongly disagree; (2) Disagree; (3) I neither disagree nor agree; (4) I agree; (5) I strongly agree. The expert agreement was defined as when the sum of (4) I agree and (5) I strongly disagree responses divided by the total of responses to each individual item were equal or greater than 0.78, as recommended by Lynn’s criteria.^20^ Items with expert agreement were maintained and those with disagreement were reformulated. The results were made available to experts and a new evaluation was performed. Reassessments were carried out until a final version was reached.

## Results

Five physicians answered the questionnaire.

The experts panel were formed by: (1) Allergist/Immunologist and pneumologist; (2) Otolaryngologists; (1) Allergist/Immunologist and (1) Otolaryngologist and Allergist/Immunologist. All experts responded to all the questionnaires.

All the questions about the outcomes (Table 1) as well as the recommendations (Table 2) of the investigation flowchart of patients with CRS reached an acceptable level of expert agreement (greater than 0.78).

**Table 1:** Expert opinion about the outcomes to be used in the evaluation of patients with chronic rhinosinusitis.

Question	Experts responses					I strongly agree + I agree
	Strongly disagree	Disagree	I neither disagree nor agree	I agree	I strongly agree	
Rate the statement according to the Likert scale: Evaluating the nasal endoscopy of a patient with chronic rhinosinusits we should use the Lund Kennedy score.	0	0	0	1	4	5/5 (100%)
Rate the statement according to the Likert scale: The SNOT-22 should be used as the disease-specific quality of life questionnaire.	0	0	0	1	4	5/5 (100%)
Rate the statement according to the Likert scale: Evaluating the computed tomography of the paranasal sinus of a patient with chronic rhinosinusits we should use the Lund-MacKay score.	0	0	0	1	4	5/5 (100%)
Rate the statement according to the Likert scale: The global visual analogue scale should be used as the subjective assessment of symptoms	0	0	0	1	4	5/5 (100%)
Rate the statement according to the Likert scale: The use of rescue medication should be used to assess adherence to treatment	0	0	0	2	3	5/5 (100%)

SNOT-22, Sinonasal Outcome Test-22; CRS, Chronic Rhinosinusitis.

**Table 2:** Experts opinion about items of the flowchart of evaluation of patients with chronic rhinosinusitis.

Question	Experts responses					I strongly agree + I agree
	Strongly disagree	Disagree	I neither disagree nor agree	I agree	I strongly agree	
The nasal endoscopy is recommended, in conjunction with history and physical examination, for patients evaluated for CRS. CT is an option to confirm the diagnosis of CRS.	0	1	0	0	4	4/5 (80%)
It is not recommended to make the diagnosis of CRS based on symptoms alone.	0	0	0	1	4	5/5 (100%)
Other diagnoses should be considered in patients with unilateral symptoms such as epistaxis, crusts or in the presence of systemic symptoms.	0	0	0	1	4	5/5 (100%)
The CT of the paranasal sinus is indicated when there is diagnostic doubt and/or; surgical indication and/or; suspected complications.	0	0	0	1	4	5/5 (100%)
Children’s diagnosed with CRS with nasal polyposis should be investigated for cystic fibrosis.	0	0	0	0	5	5/5 (100%)
Primary immunodeficiency should be considered in patients with refractory CRS.	0	0	0	0	5	5/5 (100%)
Referral to an allergist/immunologist is indicated for patients with CRS associated with otitis media, bronchitis, bronchiectasis, or pneumonia, in addition to patients who have had recurrence of symptoms after endoscopic sinus surgery. This evaluation should include serum levels of IgG, IgM, and IgA as well as an immune response to protein and polysaccharide antigens.	0	0	0	0	5	5/5 (100%)
Patients with refractory CRS associated with chronic otitis media, bronchitis, bronchiectasis, or recurrent pneumonia should be investigated for ciliary dyskinesia.	0	0	0	0	5	5/5 (100%)
A pulmonary function tests with a bronchodilator challenge should be done in all patients with CRS.	0	0	0	0	5	5/5 (100%)
Prick-test or RAST should always be done in patients with CRS.	0	1	0	0	4	4/5 (80%)
Imunological evaluation should be done in all patients with CRS.	0	0	0	0	5	5/5 (100%)
Patients with positive specific Ige for aeroallergens with a sugestive history of allergy should be refered to the allergist.	0	0	0	0	5	5/5 (100%)
NERD should always be investigated in patients with recurrent CRS with nasal poliposis and asthma.	0	0	0	0	5	5/5 (100%)
NERD can be diagnosed by a typical story of respiratory symptons indiced by nonsteroidal anti-inflammatory drugs OR by the provocation test with aspirin.	0	0	0	0	5	5/5 (100%)
CRS patients with immunoglobulin deficiency should be referred to an allergist/immunologist.	0	0	0	0	5	5/5 (100%)
Patients with refractory CRS and asthma associated with: Elevated IgE (greater than 1000 ng/mL or 400 kUI/mL) and/or elevated Aspergillus fumigatus specific IgE and/or IgG should be referred to an allergist/immunologist to investigate allergic bronchopulmonary aspergillosis.	0	0	0	1	4	5/5 (100%)

CRS, Chronic Rhinosinusitis; RAST, Radioallergosorbent test; TC, Computed Tomography; NE, Nasal Endoscopy; IgE, Immunoglobulin E; NERD, Nonsteroidal Anti-inflammatory Drug-Exacerbated Respiratory Disease.

Therefore, the flowchart was kept in its first version.

**Figure 2:**
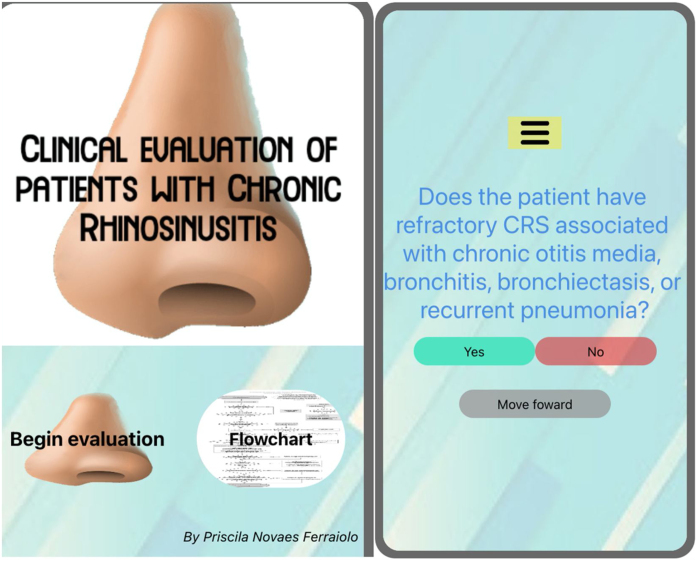
Screens from the app “Evaluations of patients with chronic rhinosinusitis”. Available on App Store: https://apps.apple.com/br/app/rinosinusite-crônica/id1545144442?l=en or on Google play: https://play.google.com/store/apps/details?id=com.gmail.priscilanferraiolo.rotinadeavaliaodepacientescomrinossinusitecrnica.

The flowchart of evaluation of patients with CRS was used as a guide for the development of a mobile application available on the App Store: https://apps.apple.com/br/app/rinosinusite-crônica/id1545144442?l=en or on Google play: https://play.google.com/store/apps/details?id=com.gmail.priscilanferraiolo.rotinadeavaliaodepacientescomrinossinusitecrnica (Fig. 2).

## Discussion

There are still many controversies regarding the correct diagnosis and the clinical evaluation of patients with symptoms of CRS as well as which outcomes should be used in the clinical practice.

With regard to the diagnosis of CRS, while some guidelines recommended performing NE to confirm the diagnosis of CRS,^16^, ^17^ others do not make it clear which is the method of choice for the diagnosis, therefore NE or CT can be performed.^14^, ^15^ All guidelines indicated that the diagnosis of CRS should not be based only on symptoms.^1^, ^14^, ^15^, ^16^, ^17^, ^18^, ^19^ Being the NE a cheaper exam and with no exposure to radiation, that can be performed during the otolaryngological exam, the consensus of experts considered appropriate to perform NE in patients with symptoms compatible with CRS, and it was decided to do the CT in cases where there is diagnostic doubt, surgical indication or suspected complications.

When analyzed the recommendations regarding allergy testing in these patients, contradictory opinions were found. Some guidelines recommended performing an allergy test for all patients with CRS,^15^, ^17^ while other recommended that this investigation can be performed, but not mandatory in all cases.^14^ A study made in São Paulo, Brazil reported that the prevalence of CRS in patients with allergic rhinitis (15.4%) was greater than the general population (3.44%).^3^ The diagnosis of allergic rhinitis can influence the prognosis of CRS patients. When investigating patients with CRS that underwent nasal surgery, those with allergic rhinitis had greater chance of needing another surgery or using dupilumabe in a five-year follow-up period.^21^ Presence of atopy was associated with younger age at the time of surgery, CRS with Nasal Polyposis (CRSwNP), asthma, eosinophilic CRS and increased severity in nasal symptom score.^22^ Patients with CRS that were treated with immunotherapy had a decreased necessity for revision surgery, interventional office visits, and intranasal and oral steroid use.^23^ As such, in the present study, the consensus of experts recommended to perform allergy tests in all patients with CRS, including: levels of serum specific IgE for Dermatophagoides pteronyssinus, Dermatophagoides farinae, blomia tropicalis and Aspergillus fumigatus.

Tanaka et al.^24^ found that 13% of CRSwNP patients and 20% of CRSwNP patients with peripheral blood eosinophilia exhibited obstructive lung dysfunction (FEV1/FVC < 70%) despite the absence of an asthma diagnosis. Another study^25^ concluded that the percent predicted forced expiratory volume in one second may be a predictor of CRSwNP recurrence after surgery. Taking this finding into account the panel of experts chose to perform a Pulmonary Function Tests (PFT) with a bronchodilator challenge in all patients with CRS to investigate the diagnosis of asthma. One guideline recommended the investigation of this comorbidity in all patients with CRS,^14^ while another considered that the investigation of asthma and the performance of a PFT should be considered in all patients with CRS with nasosinusal polyposis.^16^ PFT should be considered in all patients with CRS according to Scadding et al.^17^ and in patients with CRS and cough according to Slavin et al.^19^

The prevalence of immunodeficiency in patients with CRS is greater than in the general population. A meta-analysis concluded that the prevalence of common variable immunodeficiency, IgA deficiency and IgG deficiency was 9.4% for recurrent CRS and 18.6% for difficult-to-treat CRS.^25^ Similar results were found by Vanlerberghe and colleagues,^26^ in which 21.8% of patients with refractory sinusitis showed humoral immune disorders. Based in these findings, the expert consensus recommended referral to an allergist/immunologist to investigate immunodeficiencies in refractory CRS patients associated with other comorbidities such as chronic otitis media, bronchiectasis, or recurrent pneumonia. It was also recommended the dosage of immunoglobulins serum level (IgA, IgE, IgM and IgG serum level) and rubella serology for all patients with CRS as screening tests for immunodeficiencies. There were different recommendations regarding the investigation of immunodeficiency in patients with CRS. There were a recommendation not to investigate immunodeficiency in patients with uncomplicated CRS,^15^ as well as for investigate only in refractory cases or with other comorbidities,^16^, ^19^ or only the possibility of performing this investigation in patients with CRS with nasosinusal polyposis^17^ or even for all patients with CRS.^14^

The reliance exclusively on a history may result in either underdiagnosing or overdiagnosing of NERD hypersensitivity.^27^ One study^28^ evaluating patients with CRSwNP for the diagnosis of NERD, found that 54% of them had NERD, only 14% of those patients were diagnosed by clinical history and 40% were diagnosed by aspirin challenge test. Fifteen percent of patients with NERD didn’t know to have this comorbidity before submitted to the aspirin oral challenge, and 15% of those who self-reported having NERD didn’t have the confirmation of this diagnose after doing the aspirin oral challenge.^29^ Because of that, the expert consensus in this study chose to follow the recommendation to investigate NERD in patients with recurrent nasosinusal polyposis and asthma.^17^

Siow et al.^18^ recommended the investigation of other diagnoses, like vasculitis or tumors in cases of patients with unilateral symptoms, such as epistaxis and crusts which was the same recommendation of the expert consensus of the present study.

In the pediatric population, nasal polyps usually represents red flags indicating underlying systemic diseases, such as Cystic Fibrosis (CF), Primary Ciliary Dyskinesia (PCD) and immunodeficiencies.^30^ A study with 4044 children diagnosed with CRS found that the prevalence of cystic fibrosis was 4.1% of immune system disorder was 12.3% and 0.2% had primary ciliary dyskinesia.^31^

Individuals with CF have an incidence of CRS approaching 100%, which is often associated with nasal polyposis (6%–48%).^32^ The EPOS 2020^1^ recommended that in cases of nasal polyps among pediatric patients, investigations for CF should be performed and that sweat chloride test remains important to confirm the disease. In our study, the expert consensus recommended that children diagnosed with CRS with nasal polyposis should be investigated for cystic fibrosis with sweat chloride test.

Nasal polyps occur in approximately 18%–33% of patients with PCD, most often starting in adolescence.^33^ Other signs of PCD are chronic otitis media, chronic productive cough, and a history of recurrent respiratory infections and bronchiectasis, rhinitis, sinusitis, bronchitis and pneumonia.^33^ It was recommended by the expert panel that patients with refractory CRS associated with chronic otitis media, bronchitis, bronchiectasis or recurrent pneumonia should be investigated for ciliary dyskinesia with the sacarin test.

Patients with Allergic Bronchopulmonary Aspergillosis (ABPA) present with respiratory symptoms including poorly controlled asthma, wheeze, hemoptysis, and productive cough as well as systemic symptoms, such as fever and weight loss and can suffer recurrent exacerbations.^34^ For the diagnose of ABPA is necessary a set of minimal essential criteria: asthma, immediate cutaneous reactivity to Aspergillus fumigatus, total serum IgE > 1000 ng/mL plus one of the following: elevated specific IgE or IgG-Aspergillus Fumigatus or central bronchiectasis in the absence of distal bronchiectasis.^35^ In our experience,^36^ 31% of patients with CRS and IgE > 1000 ng/mL had the diagnose of ABPA. Therefore, it was recommended by the panel of experts that patients with refractory CRS and asthma associated with elevated IgE (greater than 1000 ng/mL or 400 kUI/mL) and/or elevated Aspergillus fumigatus-specific IgE and/or IgG should be referred to an allergist/immunologist to investigate allergic bronchopulmonary aspergillosis.

Several outcomes were chosen to be used when evaluating patients with CRS. For objective assessment of NE the expert consensus chose to use the modified Lund-Kennedy score, for the disease-specific quality of life questionnaire for CRS the SNOT-22 was the chosen one. Regarding assessment of symptoms, the use of global visual analogue scale was recommended, and for objective evaluation of the CT of the paranasal sinuses, the Lund-MacKay score was chosen. To measure adherence to treatment, the use of rescue medication was recommended by the panel of experts. All those outcomes were mentioned in the study by Soni-Jaiswal, et al.^11^

## Conclusion

With the experts panel recommendations, it was possible to establish a flowchart to guide otolaryngologists in evaluating CRS patients. These recommendations can standardize clinical routines with tests that should be requested, what comorbidities should be investigated, and which outcomes should be used in the evaluation and follow-up of patients with CRS. The mobile application with the flowchart made it easier and more accessible for it to be used by otolaryngologists on a daily routine.

## Funding

This research received no specific grant from any funding agency in the public, commercial, or not-for-profit sectors.

## Conflicts of interest

The authors declare no have conflicts of interest.
